# Predictability of Severity Scores in Patients over 70 Years Old with COVID-19 Infection: Results from a Single-Center Retrospective Observational Study

**DOI:** 10.3390/jcm15062104

**Published:** 2026-03-10

**Authors:** Andreea Magdalena Ghibu, Ionela Maniu, Victoria Birlutiu

**Affiliations:** 1 Faculty of Medicine, Lucian Blaga University of Sibiu, 550169 Sibiu, Romania; victoria.birlutiu@ulbsibiu.ro; 2 Infectious Diseases Department, Academic Emergency Hospital Sibiu, 550245 Sibiu, Romania; 3 Faculty of Sciences, Department of Mathematics and Informatics, Research Center in Informatics and Information Technology, Lucian Blaga University of Sibiu, 550024 Sibiu, Romania; 4 Research Team, Pediatric Clinical Hospital Sibiu, 550166 Sibiu, Romania

**Keywords:** Pneumonia Severity Index, CURB-65, MuLBSTA, COVID-GRAM, PADUA, age, comorbidities, mortality

## Abstract

**Background**: The elderly remain a population group at risk of developing severe forms of SARS-CoV-2 infection. Age and the prevalence of comorbidities are important risk factors for mortality related to COVID-19. Severity scores enhance medical practice, proving to be useful tools in predicting mortality. **Objectives**: The purpose of this study is to assess the predictive accuracy of the CURB-65, Pneumonia Severity Index, MuLBSTA, COVID-GRAM and PADUA scores in patients over 70 years of age diagnosed with SARS-CoV-2 infection. Methods: We performed a retrospective study between June 2023 and December 2024, analyzing demographic data, vaccination status, comorbidities, discharge status, and computed the five scores. **Results**: A total of 173 patients aged over 70 years old were included in the study. Among them, 56.06% were male with a median age of 79.72 ± 6.55. Many patients had at least one comorbidity (93.06), among which hypertension (67.63%), neurological pathology (41.61%), diabetes mellitus (28.90%) and obesity (28.32%). Tumor pathology was associated with an increased risk of death. Admission to the intensive care unit and mechanical ventilation were also risk factors for increased mortality in the study population. The area under the ROC curve (AUC) of the scores (in descending order) was: 0.914 for PADUA, 0.854 for MuLBSTA, 0.831 for PSI, 0.719 for CURB-65 and 0.703 for COVID-GRAM. **Conclusions**: All the severity scores used were significantly higher in the non-survivor group, with PSI, MuLBSTA, and PADUA scores revealing acceptable prediction ability for mortality.

## 1. Introduction

The first case of SARS-CoV-2 infection in Wuhan, China and the quick spread of this virus led to a pandemic with global implications [1,2]. From that moment until now (1 December 2025), 778,922,858 cases of COVID-19 have been reported, with 7,103,341 deaths according to the World Health Organization, and Europe and the Western Pacific region being most affected [3].

In Europe, most cases of infection reported in 2025 were recorded in Greece, Poland, and the Czech Republic. In Romania, 2200 cases were diagnosed and 20 deaths were recorded (eighth and fourth place in Europe, respectively). Most infections were reported in older people over 65, predominantly male, with or without comorbidities [3]. The continued spread of the virus and the emergence of new strains, correlating with older age, comorbidities, immunization status and the clinical status of patients on admission, highlights the necessity of using predictability scores in clinical practice [4,5]. Currently, no score has been developed for elderly patients, who remain at risk of developing severe forms of the disease and, consequently, a higher risk of mortality. Identifying a score or key parameters as predictors of mortality in this age group would significantly improve management and also provide an important base for managing future pandemics [6].

The literature identifies several scores used in the prognosis of this infection, whose accuracy has been proven or not over time. The CURB-65 score, created to assess the need for hospitalization in patients with pneumonia, is a tool often used in COVID-19 infection, including five easily assessed parameters (Appendix A Table A1) [7]. The Pneumonia Severity Index is a complex severity score that combines anamnestic elements with clinical and biological parameters, classifying patients into five risk groups (Appendix A Table A2) [8]. MuLBSTA is also an easy-to-use score that evaluates six parameters to predict the 90-day mortality of patients with COVID-19 (Appendix A Table A1) [9]. Moreover, the COVID-GRAM score stratifies patients with COVID-19 infection into three risk groups, evaluating, in addition to the above-mentioned parameters, the presence of lung damage, having been created for viral pneumonia (Appendix A Table A2) [10]. Considering the procoagulant status of the SARS-CoV-2 infection, the PADUA score evaluates 11 parameters, including age over 70 years, with a score above four pointing to being associated with an increased risk of embolism (Appendix A Table A1) [11].

These scores are useful tools for clinicians to quantify mortality in patients with this type of infection in a high-risk age group. The elderly are more susceptible to severe forms of the disease, as they often have several comorbidities and a higher risk of death from infection. The purpose of this study is to assess the performance of these scores to predict mortality in patients over 70 years old with SARS-CoV-2 infection hospitalized in our department.

## 2. Materials and Methods

### 2.1. Study Design

We conducted a retrospective, observational study in a single center among patients diagnosed with SARS-CoV-2 infection between 1 June 2023 and 31 December 2024, admitted to the Infectious Diseases Clinic of Academic Emergency Hospital Sibiu. This study was conducted in accordance with the Declaration of Helsinki and with the approval of the Ethics Committee of the “Lucian Blaga” University of Sibiu No. 4/25.07.2022, No. 26/16.06.2025 and Academic Emergency Hospital Sibiu No. 32143/22.12.2021.

### 2.2. Participants

**Figure 1:**
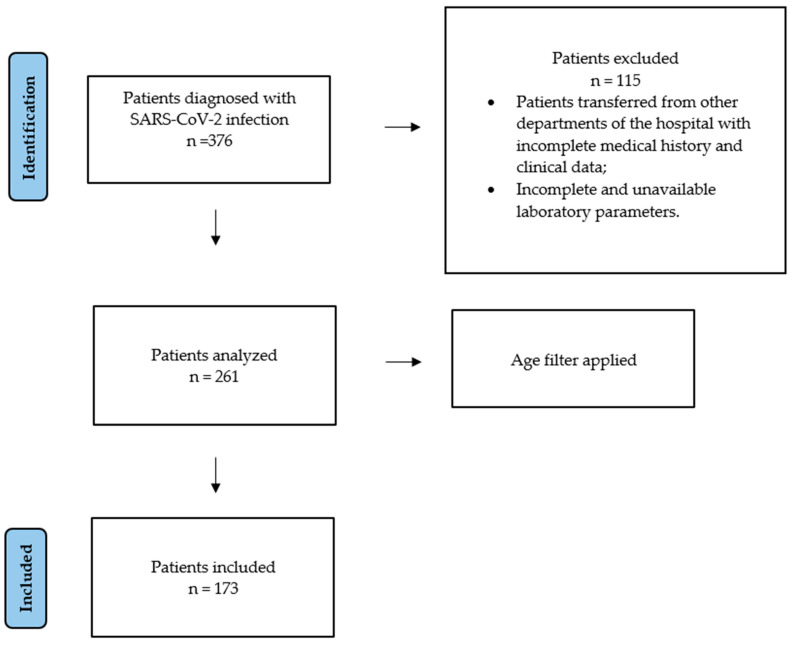
A diagram representing the process of selection.

A sample of 173 patients diagnosed with SARS-CoV-2 infection by an antigen test or RT-PCR was included in the current study, representing patients over 70 years of age (Figure 1). Patients under the age of 70, patients diagnosed with SARS-CoV-2 infection in other hospital wards but not transferred to the Infectious Diseases Department, and patients with incomplete/unavailable laboratory parameters, medical history or clinical data were excluded.

### 2.3. Data Sources

After the completion of the selection process, data were manually extracted from patient records. We took into consideration data related to symptoms, vital signs and level of consciousness evaluated in ED and recorded in patient documentation. Scores were calculated using parameters available at baseline.

Demographic data were analyzed, including place of origin, age, gender, vaccination status (including dose number), length of hospitalization, admission to the Intensive Care Unit, ventilation modes and status at discharge. Imagistically, patients were evaluated by chest X-ray and computed tomography, establishing the pulmonary involvement. The imaging data obtained during the patients’ initial assessment in the emergency department was taken into account, given that the majority of our hospitalized patients had initially passed through this department.

Comorbidities were classified into cardiovascular diseases (including hypertension, ischemic heart disease, and cardiac arrhythmias), nutritional status (based on the body mass index), metabolic disorders (e.g., type 1 and 2 diabetes mellitus), neurological disorders (e.g., sequelae of stroke, Parkinson’s disease, epilepsy), tumor pathology (e.g., colon, rectal, lung, renal neoplasm, hepatocellular carcinoma), endocrine (e.g., hypothyroidism, hyperthyroidism, autoimmune disorder), digestive (e.g., ulcer, gastritis, inflammatory bowel disease), urological, and psychiatric disorders (e.g., depression, psychosis, schizophrenia). All this information and patient history was taken from anamnesis which was performed at the admission in our department.

Furthermore, the following severity scores were calculated: CURB-65, MuLBSTA, Pneumonia Severity Index, COVID-GRAM, PADUA (Appendix A). All these data were input into a Microsoft Office Excel dataset. The primary outcome was to evaluate the accuracy of these scores regarding in-hospital mortality.

### 2.4. Statistical Analysis

The data are presented as mean and SD (standard deviation) or median and IQR (interquartile range) for continuous variables. Percentages were used for nominal variables. Comparison between survivor and non-survivor groups was assessed by a chi-square test in the case of categorial variables and the Mann–Whitney test for continuous variables. ROC (Receiver Operator Characteristic) curves were used to assess the prognostic ability of severity scores CURB-65, Pneumonia Severity Index, MuLBSTA, COVID-GRAM, and PADUA to predict mortality. Measures of diagnostic accuracy—SE (sensitivity), SP (specificity), PPV (positive predictive value), and NPV (negative predictive value)—for different cut-offs of severity scores are reported. Additionally, univariate and multivariate logistic regression models were built to assess mortality, with model 1 (M1) not adjusted for any variables, model 2 (M2) adjusted for vaccination, model 3 (M3) adjusted for tumor pathology, model 4 (M4) adjusted for ICU, model 5 (M5) adjusted for ICU and tumor pathology, model 6 (M6) adjusted for ICU, tumor pathology and vaccination, and model 7 (M7) adjusted for ICU, tumor pathology, vaccination and age. The statistical analysis was performed using R software (v. 4.5.2.) and SPSS (v. 19). The considered value for statistical significance was *p* < 0.05.

## 3. Results

Of the 173 patients included in the study, 56.06% were male, mainly from urban areas (73.41%), with the average age of 79.72 (SD = 6.55). There were 155 (89.60%) survivors and 18 (10.40%) patients died. The median age in the survivors group was 79.54 ± 6.51, and among deceased patients, it was 81.28 ± 6.9. Many patients had at least one comorbidity (93.06), 67.63% (117) patients with hypertension, 28.90% (50) with diabetes mellitus, 28.32% (49) with obesity and 41.61% (72) with previous neurological pathology. There were fewer patients with chronic kidney disease or tumor pathology. Tumor pathology was associated with a higher risk of death (*p* = 0.002). A total of 109 of the patients were vaccinated against COVID-19. Among them, 10 patients were vaccinated with a single dose, 25 with two doses and 74 patients had also received a booster shot. Admission to intensive care and initiation of mechanical ventilation were important factors in predicting mortality, being statistically significant (*p* < 0.05) (Table 1).

The central tendencies of the risk scores CURB-65, PSI, MuLBSTA, GRAM-COVID, and PADUA are presented in Table 1. The values of severity scores were significantly higher in the non-survivor group when compared to survivors (Table 2).

**Figure 2:**
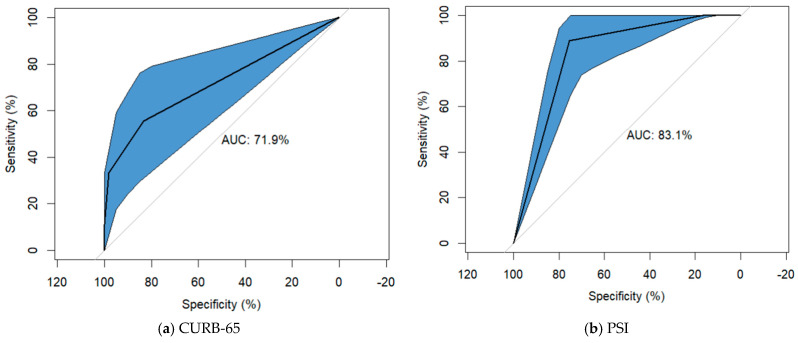

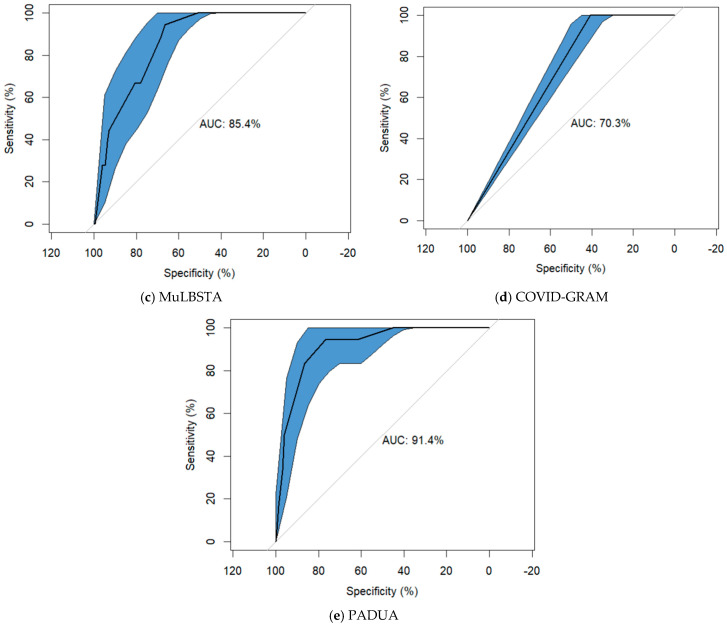
Receiver-operating characteristics (ROC) analysis.

The ROC curve analysis was performed to assess the discrimination accuracy of the severity scores. The AUC for the PADUA score was 0.914 (95%CI: [0.856–0.971], *p* < 0.001), while the sensitivity associated with the cut-off value > 4 was 94.44% (95%CI: [72.71–99.86]) and a specificity of 61.94% (95%CI: [53.80–69.61]). The MuLBSTA score had the AUC of 0.854 (95%CI: [0.785–0.922], *p* < 0.001), with the sensitivity associated with the cut-off value > 12 of 66.67% (95%CI: [40.99–86.66]) and a specificity of 78.06% (95%CI: [70.72–84.31]). In case of PSI score the AUC was 0.831 (95%CI: [0.748–0.915], *p* < 0.001), with the sensitivity associated with the cut-off value > 4 of 100.00% (95%CI: [81.47–100.00]) and a specificity of 16.77% (95%CI: [11.26–23.60]). For the CURB-65 score, the AUC was 0.719 (95%CI: [0.571–0.867], *p* = 0.002) with the sensitivity associated with the cut-off value ≥ 3 of 55.56% (95%CI: [30.76–78.47]) and a specificity of 83.23% (95%CI: [76.40–88.74]). The COVID-GRAM score had the AUC of 0.703 (95%CI: [0.608–0.799], *p* = 0.005) with the sensitivity associated with the cut-off value ≥ 3 of 100.00% (95%CI: [81.47–100.00]) and a specificity of 40.65% (95%CI: [32.84–48.82]) (Table 3, Figure 2).

The results of unadjusted analysis, showed that all five scores were significantly associated with mortality. After adjusting for ICU admission, tumor pathology, and vaccination status, the PADUA (aOR 2.528), PSI (aOR 1.031), and MuLBSTA (aOR 1.365) scores remained independent predictors of death (Table 4).

## 4. Discussion

Our study compares five severity scores in a cohort of patients with SARS-CoV-2 infection in terms of in hospital mortality. To our knowledge, the predictive value of the CURB-65 score has been evaluated before in the Romanian population; however, it has not been evaluated for Pneumonia Severity Index, MuLBSTA, COVID-GRAM, and PADUA. This study is a retrospective, observational study that evaluates the predictability of those scores among patients over 70 years old with the COVID-19 infection and admitted to the Infectious Diseases Department of the Academic Emergency Hospital Sibiu. A mortality rate of 10.4% was obtained, which is lower than in previous reports that analyzed all age groups [12,13,14]. The antiviral therapies and vaccination of patients could decrease the fatality rate. However, a significant number of patients, representing 36.99% of the study group, did not opt for vaccination, most of them coming from urban areas, which leads to a continued vulnerability in this group. Males remain the most affected, correlating with data from the literature [15,16,17,18,19]. Furthermore, studies showed that the use of antibiotics did not significantly improve the risk of death, independently of the timing of patients’ referral [20]. Previous exposure to antibiotics causes disturbances in the gut microbiota, as well as disruptions in the immune response to vaccination. McAlister et al. concluded that patients who received antibiotics in the last three months developed severe forms of the disease with poor 30-day outcomes after the patient was diagnosed with the COVID-19 infection [21,22].

The patients included in the study presented various pathologies, hypertension (67.63%), neurological pathology (41.61%), diabetes mellitus (28.90%) and obesity (28.32%). Hypertension was also the most common comorbidity identified in patients with COVID-19 [23]. Tumor pathology and chronic kidney disease were identified in a lower proportion, which is in accordance with the literature on this subject [24,25,26,27,28]. Among patients with tumor pathology, almost half died, which emphasizes the fact that immunodeficiency is a negative prognostic factor. Rodriguez-Nava et al. highlight, in a cohort of 313 patients, that 85% of the patients analyzed had at least two significant comorbidities, a finding that was also observed in our study [29]. Furthermore, admission to the intensive care unit (77.77% vs. 5.80%) and mechanical ventilation were negative prognostic factors and were also associated with an increased risk of mortality.

In our study, the CURB-65 score achieved an AUC of 0.719 (95% CI: [0.571–0.867], *p* = 0.002), a high specificity of 83.2% at a cut-off point of 3, but the lowest sensitivity of 55.5%. Our results are in line with those obtained by Su et al. This could be explained by the fact that although it includes age as a risk factor, it does not assess comorbidities. Although it has the lowest sensitivity, the CURB-65 score has the benefit of including a single laboratory parameter, which makes it easier to calculate [30]. A similar AUROC value was observed in the study conducted by Knight et al. on a cohort of 35,463 patients with a mean age of 73 years [31]. Citu et al. demonstrated good predictability of the CURB-65 score (AUC: 0.801; 95% CI: [0.681–0.922], *p* < 0.001) [32] regarding mortality, with a similar result obtained by Amr Elmoheen et al. in a study involving 1181 patients [8,33]. Its mortality predictive value is also preserved at 30 days, as highlighted by Lucijanić et al. [34].

In a study conducted on a cohort of 10,238 patients, comparing the CURB-65, PSI, MuLBSTA, and qSOFA scores, Arturo et al. highlighted the superiority of the PSI score in predicting mortality, which can also be observed in our study [33,35]. The performance of the PSI score in relation to the CURB-65 score was also highlighted in the study performed by Bradley et al. on a group of 8081 patients in 2022, also achieving slightly better ROC curve by associating d-dimers and procalcitonin [36,37]. Felippe Lazar Neto et al. in 2021 also highlighted, in a comparative analysis of PSI, CURB, CURB-65, COVID-GRAM, IDSA/ATS Minor Criteria, qSOFA, SCAP, SMART-COP, REA-ICU, CALL, and 4C scores, the superiority of PSI (AUC: 0.79; 95% CI: [0.77–0.82]) with a sensitivity of 0.90 (95% CI: [0.86–0.93]) and a specificity of 0.49 (95% CI: [0.46–0.52]). This could be a future direction to explore and verify the predictability of these scores beyond 30 days, at six months or one year. He also emphasized that the presence of comorbidities and age remain two strong independent risk factors for mortality in SARS-CoV-2 infection [38].

With a good prediction of 90-day mortality [9,27] at a cut-off point of 12 [39], among patients with the COVID-19 infection and aged over 70 years old from our study, the MuLBSTA score proved to be a good predictor of mortality with statistical significance and an AUC of 0.854 (95%CI: [0.785–0.922], *p* < 0.001). This score additionally quantifies multilobar involvement and the bacterial co-infections, the elderly patients being more susceptible to severe pulmonary involvement. Also, among the cardiac comorbidities, the presence of hypertension is an individually quantified parameter, this condition being the most common in the studied cohort. Cheng et al. reported similar outcomes, concluding that this score is a highly effective tool for assessing mortality risk, providing superior results than CURB-65 and APACHE II score, which is also a reliable tool for assessing disease severity [40]. Furthermore, Preti et al. highlight that the MuLBSTA score preserves its predictive value [41] in a study conducted on a group of 431 patients regarding 28-day mortality prediction using PSI, MuLBSTA, and CURB-65 scores,.

The COVID-GRAM score proved to be a good predictor of mortality among elderly patients, assessing from 0 to 5 comorbidities, including hypertension and diabetes, two important groups of comorbidities in our study, as well as an LDH assessment, which is an important marker for quantifying severity. Thus, in our study, at a cut-off point of 89 corresponding to the high-risk group, the COVID-GRAM score shows 100% sensitivity for mortality (95%CI: [81.47–100.00]). Esteban Ronda et al. also obtained good predictability of this score in a group of 208 patients with a median age of 63 years, reporting high sensitivities for CURB-65, COVID-GRAM and PSI [10]. Moreover, high AUC for the Pneumonia Severity Index score and MuLBSTA score (0.831 and 0.853) was obtained, but a similar sensitivity to COVID-GRAM. The accuracy of the COVID-GRAM score was also highlighted by Martha A. Mendoza-Hernandez et al., in a study of 514 patients, who analyzed seven severity scores in a center with high mortality due to COVID-19 infection, applied at baseline and at two, four, six, and eight days of hospitalization. It was also pointed out that the COVID-GRAM and MuLBSTA scores maintained their predictive capacity regardless of when they were applied, which gives these scores predictive stability. The predictive value of the PSI score increased, which can be explained by the variability of the clinical parameters used, which change depending on the time of hospitalization. Thus, the temporal variability of clinical parameters can significantly influence the predictive effectiveness of each score [42].

Regarding procoagulant status, the PADUA score achieved good results, being a good predictor of mortality due to possible thrombotic events, achieving, in our study a good sensitivity of 94.4% at a cut-off point ≥ 4 and an AUC of 0.914 (95%CI: [0.856–0.971], *p* < 0.001). The literature reports an increased mortality at this cut-off point at a rate up to 63% and highlights the importance of prophylactic anticoagulation in patients with severe/critical forms of the disease, who are at high risk of developing thrombotic events [43]. Furthermore, using the PADUA score helped to achieve lower risk of venous thromboembolism through effective anticoagulation, which has decreased the number of deaths caused by thrombotic events, especially during the cytokine storm that induces a coagulation cascade, through insufficient control of anti-inflammatory factors over pro-inflammatory cytokines, leading to thrombosis events [44,45,46].

However, our study has several limitations. First, it is a retrospective study conducted in a single center with a relatively small number of patients enrolled. The small number of events (18 deaths) limit the robustness of the conclusions. Although the scores had good discriminative ability (PADUA AUC: 0.914, MuLBSTA AUC: 0.854, PSI AUC: 0.831, CURB-65 AUC: 0.719, COVID-GRAM AUC: 0.703), and the optimism-corrected AUC results of bootstrapping analysis (internal validation with 1000 bootstrap resamples) remained high (corrected AUC for PADUA: 0.915, MuLBSTA: 0.854, PSI: 0.832, CURB-65 AUC: 0.718, COVID-GRAM AUC: 0.703) indicating internal stability, the score’s performance should be confirmed in larger, multicenter cohort with different patient populations and varying mortality risks. Also, due to the small number of events, the identified cut-off may lack stability across different clinical cohorts. The high sensitivity and relatively poor specificity of several models (PSI, COVID-GRAM) introduce a substantial risk of false-positive stratification. While these tools are valuable for identifying patients requiring increased clinical vigilance, their use in the geriatric population should be adjunctive adjuvant. Clinical decision-making should integrate these numerical scores with bedside clinical judgment, individual goals of care, multidisciplinary assessment of the patient’s overall prognosis. Another limitation is that these scores were calculated only at the baseline, which does not provide a perspective on the effectiveness of these scores over time. Last but not least, the lack of comparison with pandemic waves and highlighting the predictability of these scores based on the circulating variant of the virus in order to identify possible differences in the predictive accuracy of these scores represents another limitation.

## 5. Conclusions

All scores analyzed in this study proved to be effective in the study group. However, the Pneumonia Severity Index, MuLBSTA and PADUA scores revealed acceptable prediction ability for mortality among patients hospitalized with COVID-19 and aged over 70 years old. The complexity of the parameters assessed by the scores analyzed led to greater accuracy in predicting mortality. However, the simplicity and short time required to complete these scores are important factors that influence their use in clinical practice. Future research should focus on the prospective multi-center validation of the scores in larger (geriatric) cohorts to establish more accurate clinical cut-offs. Additionally, incorporating viral sequencing, additional measures, and longitudinal data through machine learning algorithms (to develop dynamic risk models that update based on laboratory trends) may enhance the specificity of these models, and may reduce the risks of complications while improving personalized care for the patients.

## Figures and Tables

**Table 1:** Characteristics of patients.

Characteristics		All (n = 173)	Survivors (n = 155)	Died (n = 18)	*p*-Value
Gender	M	97 (56.06)	85 (54.83)	12 (66.66)	0.339
	F	76 (43.93)	70 (45.16)	6 (33.33)	
Age	mean ± SD	79.72 ± 6.55	79.54 ± 6.51	81.28 ± 6.91	0.293
Environment	U	127 (73.41)	114 (73.54)	13 (72.22)	0.904
	R	46 (26.58)	41 (26.45)	5 (27.77)	
COVID-19 vaccination	No	64 (36.99)	55 (35.48)	9 (50.50)	0.227
	Yes	109 (63.00)	100 (64.51)	9 (50.50)	
Hypertension	No	56 (32.36)	47 (30.32)	9 (50.50)	0.091
	Yes	117 (67.63)	108 (69.67)	9(50.50)	
Weight	Normal	86 (49.71)	73 (47.09)	13 (72.22)	0.129
	Overweight	38 (21.96)	36 (23.22)	2 (11.11)	
	Obesity	49 (28.32)	46 (29.67)	3 (16.66)	
Diabetes mellitus	No	123 (71.09)	110 (70.96)	13 (72.22)	0.912
	Yes	50 (28.90)	45 (29.03)	5 (27.77)	
Chronic kidney disease	No	149 (86.12)	132 (85.16)	17 (94.44)	0.281
	Yes	24 (13.87)	23 (14.83)	1 (5.55)	
Prior neurological condition	No	101 (58.38)	89 (57.41)	12 (66.66)	0.451
	Yes	72 (41.61)	66 (42.58)	6 (33.33)	
Tumor pathology	No	148 (85.54)	137 (88.38)	11 (61.11)	0.002
	Yes	25 (14.45)	18 (11.61)	7 (38.88)	
ICU admission	No	150 (86.70)	146 (94.19)	4 (22.22)	0.000
	Yes	23 (13.29)	9 (5.80)	14 (77.77)	
Mechanic ventilation	No	165 (95.37)	153 (98.70)	12 (66.66)	0.000
	Yes	8 (4.62)	2 (1.29)	6 (33.33)	
Hospitalization days	median (IQR)	7 (6; 10)	7 (6; 10)	8 (4; 11)	0.785

**Table 2:** Comparison of severity scores.

Severity Scores	All (n = 173)	Survivors (n = 155)	Died (n = 18)	*p*-Value
	Mean ± SD; Median (IQR)			
CURB-65	2.27 ± 0.60 2.00 (2.00; 2.00)	2.18 ± 0.45 2.00 (2.00; 2.00)	3.00 ± 1.08 3.00 (2.00; 4.00)	0.000
PSI	118.93 ± 30.41 113.00 (99.00; 135.00)	114.26 ± 25.93 111.00 (96.00; 130.00)	159.17 ± 36.75 155.50 (134.50; 190.50)	0.000
MuLBSTA	9.27 ± 3.82 9.00 (7.00; 12.00)	8.77 ± 3.61 8.00 (7.00; 11.00)	13.56 ± 2.71 13.00 (11.00; 17.00)	0.000
COVID-GRAM	56.60 ± 32.29 60.20 (29.20; 84.20)	53.11 ± 31.96 54.10 (24.10; 79.10)	86.59 ± 15.14 93.58 (73.58; 96.58)	0.000
PADUA	3.60 ± 2.13 3.00 (2.00; 5.00)	3.23 ± 1.86 3.00 (2.00; 4.00)	6.78 ± 1.70 6.50 (6.50; 8.50)	0.000

SD—standard deviation; IQR—interquartile range.

**Table 3:** Sensitivity, specificity, positive predictive value, and negative predictive value of the severity scores.

Severity Scores	Cut-Off Value	SE	SP	PPV	NPV
CURB-65	3	55.56% (30.76–78.47%)	83.23% (76.40–88.74%)	27.78% (18.28–39.81%)	94.16% (90.54–96.45%)
PSI	4	100.00% (81.47–100.00%)	16.77% (11.26–23.60%)	12.24% (11.51–13.02%)	100.00% (86.77–100.00%)
MuLBSTA	12	66.67% (40.99–86.66%)	78.06% (70.72–84.31%)	26.09% (18.50–35.44%)	95.28% (91.26–97.50%)
COVID-GRAM	3	100.00% (81.47–100.00%)	40.65% (32.84–48.82%)	16.36% (14.66–18.23%)	100.00% (94.31–100.00%)
PADUA	4	94.44% (72.71–99.86%)	61.94% (53.80–69.61%)	22.37% (18.63–26.61%)	98.97% (93.44–99.85%)

SE—sensitivity, SP—specificity, PPV—positive predictive value, NPV—negative predictive value.

**Table 4:** Univariate and multivariate logistic regression, adjusted for vaccination, tumor pathology, ICU, and age.

Severity Scores	OR M1	aOR M2	aOR M3	aOR M4	aOR M5	aOR M6	aOR M7
CURB-65	4.587 (2.377–9.624) 0.000	4.806 (2.448–10.329) 0.000	4.534 (2.266–10.105) 0.000	2.088 (0.890–5.291) 0.104	2.181 (0.905–5.672) 0.094	2.376 (0.960–6.309) 0.071	2.420 (0.965–6.592) 0.071
PSI	1.047 (1.029–1.071) 0.000	1.052 (1.032–1.077) 0.000	1.045 (1.026–1.069) 0.000	1.029 (1.007–1.054) 0.012	1.027 (1.005–1.053) 0.022	1.031 (1.007–1.059) 0.015	1.031 (1.007–1.059) 0.015
MuLBSTA	1.449 (1.243–1.733) 0.000	1.435 (1.231–1.719) 0.000	1.436 (1.225–1.731) 0.000	1.382 (1.122–1.762) 0.004	1.367 (1.110–1.743) 0.006	1.365 (1.105–1.749) 0.007	1.362 (1.103–1.746) 0.007
COVID- GRAM	1.040 (1.019–1.068) 0.001	1.040 (1.019–1.068) 0.001	1.036 (1.014–1.065) 0.005	1.025 (1.000–1.051) 0.039	1.021 (0.996–1.049) 0.097	1.021 (0.996–1.049) 0.101	1.021 (0.996–1.049) 0.098
PADUA	2.270 (1.698–3.280) 0.000	2.258 (1.686–3.268) 0.000	2.456 (1.743–3.786) 0.000	2.147 (1.491–3.427) 0.000	2.534 (1.612–4.555) 0.000	2.528 (1.608–4.534) 0.000	2.521 (1.600–4.533) 0.000

Results are presented as OR (odd ratio), 95% CI (confidence intervals), *p*-value, M1—model 1 not adjusted for any variables, M2—model 2 adjusted for vaccination, M3—model 3 adjusted for tumor pathology, M4—model 4 adjusted for ICU, M5—model 5 adjusted for ICU and tumor pathology, M6—model 6 adjusted for ICU, tumor pathology and vaccination, M7—model 7 adjusted for ICU, tumor pathology, vaccination and age.

## Data Availability

Data are contained within the article.

## Abbreviations

The following abbreviations are used in this manuscript:

SARS-CoV-2	Severe acute respiratory syndrome coronavirus 2
COVID-19	Coronavirus disease 2019
RT-PCR	Real-Time Polymerase Chain Reaction
LDH	Lactate dehydrogenase
BMI	Body Mass Index
SBP	Systolic Blood Pressure
DBP	Diastolic Blood Pressure
ICU	Intensive Care Unit
PSI	Pneumonia Severity Index

## Appendix A

**Table A1:** CURB-65 Score, MuLBSTA score, and PADUA Score.

Characteristics	Yes	CURB-65	MulBSTA	PADUA
Confusion	+1	✓		
Blood urea ≥ 19 mg/dL	+1	✓		
Respiratory rate ≥ 30 breaths/min	+1	✓		
SBP < 90 mmHg, DBP ≤ 60 mmHg	+1	✓		
Age		≥65 years +1	≥60 years +2	≥70 years +1
Multilobar involvement	+5		✓	
Lymphocyte count ≤ 0.8 × 109/L	+4		✓	
Bacterial coinfection	+4		✓	
Smoking status			✓	
Active smoker	+3			
Prior smoker	+2			
History of hypertension	+2		✓	
Active cancer	+3			✓
History of pulmonary embolism/Deep vein thrombosis	+3			✓
Limited mobility	+3			✓
Diagnosed thrombophilia	+3			✓
Trauma/Recent surgery < 1 month	+2			✓
**Heart/respiratory failure**	+1			✓
Heart attack/Ischemic stroke	+1			✓
Acute infection and/or rheumatic diseases	+1			✓
Obesity (BMI ≥ 30 kg/m^2^)	+1			✓
Hormone therapy	+1			✓

**Table A2:** Pneumonia Severity Index and COVID-GRAM score.

Characteristics	Yes	Pneumonia Severity Index ^1^	COVID-GRAM ^2^
Age		✓	✓
Male	Years	✓	
Female	Years-10	✓	
Nursing home resident	Years + 10	✓	
Tumor/History of tumors	+30	✓	✓
Liver disease	+20	✓	
Congestive heart failure	+10	✓	
Cerebrovascular disease	+10	✓	
Kidney disease	+10	✓	
Altered mental status	+20	✓	✓
Respiratory rate ≥ 30 breaths/min	+20	✓	
Systolic blood pressure < 90 mmHg	+20	✓	
Temperature < 35 °C sau > 40 °C	+15	✓	
Heart rate > 125 bpm	+10	✓	
Arterial pH < 7.35	+30	✓	
Blood Urea ˃ 30 mg/dL	+20	✓	
Sodium < 130 mmol/L	+20	✓	
Glucose > 250 mg/dL	+10	✓	
Hematocrit < 30%	+10	✓	
Partial pressure of oxygen < 60 mmHg	+10	✓	
Abnormalities on chest X-ray	+10	Pleural effusion	✓
Hemoptysis			✓
Dyspnea			✓
Number of comorbidities:0–5 *			✓
Neutrophil-Lymphocyte Ratio			✓
LDH			✓
Direct Bilirubin			✓

^1^ Five risk groups: I—under the age of 50, with no associated pathology and normal parameter values, II—under 70 points, III—71–90 points, IV—91–130 points, V—more than 131 points. ^2^ Three risk groups: Low-risk group: <1.7%, Medium risk group: 1.7–40.4%, High risk group: ≥40.4%. * Comorbidities: COPD, Hypertension, Diabetes mellitus, Heart disease, Chronic kidney disease, Tumors, Cerebrovascular disease, Hepatitis B, Immunodeficiency.

## References

[1] Xie J., Wu W., Li S., Hu Y., Hu M., Li J., Yang Y., Huang T., Zheng K., Wang Y. (2020). Clinical characteristics and outcomes of critically ill patients with novel coronavirus infectious disease (COVID-19) in China: A retrospective multicenter study. Intensive Care Med..

[2] Huang C., Wang Y., Li X., Ren L., Zhao J., Hu Y., Zhang L., Fan G., Xu J., Gu X. (2020). Clinical features of patients infected with 2019 novel coronavirus in Wuhan, China. Lancet.

[3] WHO WHO Coronavirus Disease (COVID-19) Dashboard. https://covid19.who.int.

[4] Bradley P., Frost F., Tharmaratnam K., Wootton D.G., NW Collaborative Organization for Respiratory Research (2020). Utility of established prognostic scores in COVID-19 hospital admissions: Multicenter prospective evaluation of CURB-65, NEWS2 and qSOFA. BMJ Open Respir. Res..

[5] Lombardi Y., Azoyan L., Szychowiak P., Bellamine A., Lemaitre G., Bernaux M., Daniel C., Leblanc J., Riller Q., Steichen O. (2021). External Validation of Prognostic Scores for COVID-19: A Multicenter Cohort Study of Patients Hospitalized in Greater Paris University Hospitals. Intensive Care Med..

[6] Hokenek H.D., Julide S.K. (2023). Comparison of scoring systems’ mortality prediction ability in COVID-19 intensive care patients over 80 years of age. Ann. Clin. Anal. Med..

[7] Oliva A., Borrazzo C., Mascellino M.T., Curtolo A., Al Ismail D., Cancelli F., Galardo G., Bucci T., Ceccarelli G., d’Ettorre G. (2021). CURB-65 plus hypoalbuminemia: A new score system for prediction of the in-hospital mortality risk in patients with SARS-CoV-2 pneumonia. Infez. Med..

[8] Elmoheen A., Abdelhafez I., Salem W., Bahgat M., Elkandow A., Tarig A., Arshad N., Mohamed K., Al-Hitmi M., Saad M. (2021). External Validation and Recalibration of the CURB-65 and PSI for Predicting 30-Day Mortality and Critical Care Intervention in Multiethnic Patients with COVID-19. Int. J. Infect. Dis..

[9] George R., Mehta A.A., Paul T., Sathyapalan D.T., Haridas N., Kunoor A., Ravindran G.C. (2022). Validation of MuLBSTA score to derive modified MuLB score as mortality risk prediction in COVID-19 infection. PLoS Glob. Public Health.

[10] Esteban Ronda V., Ruiz Alcaraz S., Ruiz Torregrosa P., Giménez Suau M., Nofuentes Pérez E., León Ramírez J.M., Andrés M., Moreno-Pérez Ó., Candela Blanes A., Gil Carbonell J. (2021). Application of validated severity scores for pneumonia caused by SARS-CoV-2. Med. Clin. (Engl. Ed.).

[11] Zeng D.X., Xu J.L., Mao Q.X., Liu R., Zhang W.Y., Qian H.Y., Xu L. (2020). Association of Padua prediction score with in-hospital prognosis in COVID-19 patients. QJM Int. J. Med..

[12] Citu C., Gorun F., Motoc A., Sas I., Gorun O.M., Burlea B., Tuta-Sas I., Tomescu L., Neamtu R., Malita D. (2022). The Predictive Role of NLR, d-NLR, MLR, and SIRI in COVID-19 Mortality. Diagnostics.

[13] Macedo A., Gonçalves N., Febra C. (2021). COVID-19 Fatality Rates in Hospitalized Patients: Systematic Review and Meta-Analysis. Ann. Epidemiol..

[14] Toker İ., Kılınç-Toker A., Turunç-Özdemir A., Altuntaş M. (2022). Comparison of CURB-65 Pneumonia Severity Score, Quick COVID-19 Severity Index, and Brescia-COVID Respiratory Severity Scale in Emergently Hospitalized COVID-19 Patients with Pneumonia. Infect. Dis. Clin. Microbiol..

[15] Ocho K., Hagiya H., Hasegawa K., Fujita K., Otsuka F. (2022). Clinical Utility of 4C Mortality Scores among Japanese COVID-19 Patients: A Multicenter Study. J. Clin. Med..

[16] Eldaboosy S., Almoosa Z., Saad M., Al Abdullah M., Farouk A., Awad A., Mahdy W., Abdelsalam E., Nour S.O., Makled S. (2022). Comparison Between Physiological Scores SIPF, CURB-65, and APACHE II as Predictors of Prognosis and Mortality in Hospitalized Patients with COVID-19 Pneumonia: A Multicenter Study, Saudi Arabia. Infect. Drug Resist..

[17] Sprockel J.J., Murcia A.L., Díaz M.C., Rios L.F., Quirós O.I., Parra J.E. (2024). Performance of APACHE II, SOFA, and CURB-65 for death prognosis in COVID-19 critical patients: A prospective cohort study. Acta Colomb. Cuid. Intensivo.

[18] Innocenti F., De Paris A., Lagomarsini A., Pelagatti L., Casalini L., Gianno A., Montuori M., Bernardini P., Caldi F., Tassinari I. (2022). Stratification of patients admitted for SARS-CoV2 infection: Prognostic scores in the first and second wave of the pandemic. Intern. Emerg. Med..

[19] Ahmed A., Alderazi S.A., Aslam R., Barkat B., Barker B.L., Bhat R., Cassidy S., E Crowley L., Dosanjh D.P., Ebrahim H. (2022). Utility of severity assessment tools in COVID-19 pneumonia: A multicentre observational study. Clin. Med..

[20] Stoichitoiu L.E., Pinte L., Ceasovschih A., Cernat R.C., Vlad N.D., Padureanu V., Sorodoc L., Hristea A., Purcarea A., Badea C. (2022). In-Hospital Antibiotic Use for COVID-19: Facts and Rationales Assessed through a Mixed-Methods Study. J. Clin. Med..

[21] Cheung K.S., Lam L.K., Zhang R., Ooi P.H., Tan J.T., To W.P., Hui C.H., Chan K.H., Seto W.K., Hung I.F.N. (2022). Association between Recent Usage of Antibiotics and Immunogenicity within Six Months after COVID-19 Vaccination. Vaccines.

[22] McAlister F.A., Lin M., Youngson E., Lethebe B.C., Leslie M. (2025). Prior antibiotic exposure is associated with worse outcomes in adults with COVID-19. Infect. Dis..

[23] Ucan E.S., OzgenAlpaydin A., Ozuygur S.S., Ercan S., Unal B., Sayiner A.A., Ergan B., Gokmen N., Savran Y., Kilinc O. (2021). DEU COVID StudyGroup. Pneumonia severity indices predict prognosis in coronavirus disease-2019. Respir. Med. Res..

[24] Khari S., Salimi Akin Abadi A., Pazokian M., Yousefifard M. (2022). CURB-65, qSOFA, and SIRS Criteria in Predicting In-Hospital Mortality of Critically Ill COVID-19 Patients; a Prognostic Accuracy Study. Arch. Acad. Emerg. Med..

[25] Carriel J., Muñoz-Jaramillo R., Bolaños-Ladinez O., Heredia-Villacreses F., Menéndez-Sanchón J., Martin-Delgado J., en representación del grupo de investigadores COVID-EC (2022). CURB-65 como predictor de mortalidad a 30 días en pacientes hospitalizados con COVID-19 en Ecuador: Estudio COVID-EC CURB-65 as a predictor of 30-day mortality in patients hospitalized with COVID-19 in Ecuador: COVID-EC study. Rev. Clin. Esp..

[26] Gruyters I., De Ridder T., Bruckers L., Geebelen L., Gharmaoui S., Callebaut I., Vandenbrande J., Berends N., Dubois J., Stessel B. (2022). Predictive value of serial evaluation of the Sequential Organ Failure Assessment (SOFA) score for intensive care unit mortality in critically ill patients with COVID-19: A retrospective cohort study. Anaesthesiol. Intensive Ther..

[27] Boesing M., Lüthi-Corridori G., Büttiker D., Hunziker M., Jaun F., Vaskyte U., Brändle M., Leuppi J.D. (2024). The Predictive Performance of Risk Scores for the Outcome of COVID-19 in a 2-Year Swiss Cohort. Biomedicines.

[28] Costa Mello V.L., do Basil P.E.A.A. (2024). Fully independent validation of eleven prognostic scores predicting progression to critically ill condition in hospitalized patients with COVID-19. Braz. J. Infect. Dis. Off. Publ. Braz. Soc. Infect. Dis..

[29] Rodriguez-Nava G., Yanez-Bello M.A., Trelles-Garcia D.P., Chung C.W., Friedman H.J., Hines D.W. (2021). Performance of the quick COVID-19 severity index and the Brescia-COVID respiratory severity scale in hospitalized patients with COVID-19 in a community hospital setting. Int. J. Infect. Dis..

[30] Su Y., Tu G.W., Ju M.J., Yu S.J., Zheng J.L., Ma G.G., Liu K., Ma J.F., Yu K.H., Xue Y. (2020). Comparison of CRB-65 and quick sepsis-related organ failure assessment for predicting the need for intensive respiratory or vasopressor support in patients with COVID-19. J. Infect..

[31] Knight S.R., Ho A., Pius R., Buchan I., Carson G., Drake T.M., Dunning J., Fairfield C.J., Gamble C., Green C.A. (2020). ISARIC4C investigators. Risk stratification of patients admitted to hospital with COVID-19 using the ISARIC WHO Clinical Characterisation Protocol: Development and validation of the 4C Mortality Score. BMJ.

[32] Citu C., Gorun F., Motoc A., Ratiu A., Gorun O.M., Burlea B., Neagoe O., Citu I.M., Rosca O., Bratosin F. (2022). Evaluation and Comparison of the Predictive Value of 4C Mortality Score, NEWS, and CURB-65 in Poor Outcomes in COVID-19 Patients: A Retrospective Study from a Single Center in Romania. Diagnostics.

[33] Artero A., Madrazo M., Fernández-Garcés M., Miguez A.M., García A.G., Vieitez A.C., Guijarro E.G., Aizpuru E.M.F., Gómez M.G., Manrique M.A. (2021). Severity Scores in COVID-19 Pneumonia: A Multicenter, Retrospective, Cohort Study. J. Gen. Intern. Med..

[34] Lucijanić M., Piskač Živković N., Režić T., Durlen I., Stojić J., Jurin I., Šakota S., Filipović D., Kurjaković I., Jordan A. (2023). The performance of the WHO COVID-19 severity classification, COVID-GRAM, VACO Index, 4C Mortality, and CURB-65 prognostic scores in hospitalized COVID-19 patients: Data on 4014 patients from a tertiary center registry. Croat. Med. J..

[35] Wang L., Lv Q., Zhang X., Jiang B., Liu E., Xiao C., Yu X., Yang C., Chen L. (2020). The utility of MEWS for predicting the mortality in the elderly adults with COVID-19: A retrospective cohort study with comparison to other predictive clinical scores. PeerJ.

[36] Bradley J., Sbaih N., Chandler T.R., Furmanek S., Ramirez J.A., Cavallazzi R. (2022). Pneumonia Severity Index and CURB-65 Score Are Good Predictors of Mortality in Hospitalized Patients With SARS-CoV-2 Community-Acquired Pneumonia. Chest.

[37] Ghibu A.M., Maniu I., Birlutiu V. (2026). Severity Scores in SARS-CoV-2 Infection—A Comprehensive Bibliometric Review and Visualization Analysis. Epidemiologia.

[38] Lazar Neto F., Marino L.O., Torres A., Cilloniz C., Marchini J.F.M., de Alencar J.C.G., Palomeque A., Albacar N., Neto R.A.B., Souza H.P. (2021). Community-acquired pneumonia severity assessment tools in patients hospitalized with COVID-19: A validation and clinical applicability study. Clin. Microbiol. Infect..

[39] Preetam M., Anurag A. (2021). MuLBSTA score in COVID-19 pneumonia and prediction of 14-day mortality risk: A study in an Indian cohort. J. Fam. Med. Prim. Care.

[40] Cheng P., Wu H., Yang J., Song X., Xu M., Li B., Zhang J., Qin M., Zhou C., Zhou X. (2021). Pneumonia scoring systems for severe COVID-19: Which one is better. Virol. J..

[41] Preti C., Biza R., Novelli L., Ghirardi A., Conti C., Galimberti C., Della Bella L., Memaj I., Di Marco F., Cosentini R. (2022). Usefulness of CURB-65, pneumonia severity index and MuLBSTA in predicting COVID-19 mortality. Monaldi Arch. Chest Dis..

[42] Mendoza-Hernandez M.A., Hernandez-Fuentes G.A., Sanchez-Ramirez C.A., Rojas-Larios F., Guzman-Esquivel J., Rodriguez-Sanchez I.P., Martinez-Fierro M.L., Cardenas-Rojas M.I., De-Leon-Zaragoza L., Trujillo-Hernandez B. (2024). Time-dependent ROC curve analysis to determine the predictive capacity of seven clinical scales for mortality in patients with COVID-19: Study of a hospital cohort with very high mortality. Biomed. Rep..

[43] Abutaleb A., Nathan S. (2021). COVID-19 infection-associated coagulopathy: Pathophysiology and clinical implications. Interv. Neuroradiol..

[44] Rotty L., Hendratta C., Damay V., Haroen H., Lasut P.F., Wariki W.M. (2023). Padua Score and Coagulopathy Parameters on Survival of COVID-19 Patients at Prof Dr. R. D. Kandou General Hospital Manado. Open Access Maced. J. Med Sci..

[45] Casey K., Iteen A., Nicolini R., Auten J. (2020). COVID-19 pneumonia with hemoptysis: Acute segmental pulmonary emboli associated with novel coronavirus infection. Am. J. Emerg. Med..

[46] Xie Y., Wang X., Yang P., Zhang S. (2020). COVID-19 complicated by acute pulmonary embolism. Radiol. Cardiothorac. Imaging.

